# The Role of Perspective Taking and Self-Control in a Preventive Intervention Targeting Childhood Disruptive Behavior

**DOI:** 10.1007/s10802-020-00761-1

**Published:** 2021-01-13

**Authors:** Karlijn Nijhof, Lysanne W. te Brinke, Urdur Njardvik, Juliette M. Liber

**Affiliations:** 1 Department of Developmental Psychology, Utrecht University, Utrecht, Netherlands; 2 Department of Psychology, University of Iceland, Reykjavik, Iceland

**Keywords:** Perspective taking, Self-control, Disruptive behavior, Social-cognitive, Preventive intervention

## Abstract

Prevention studies typically focus on outcome variables such as reductions in problem behavior, rather than targeted factors (e.g., cognitions), or the relation between change in targeted factors and outcomes. Therefore, the current study examined the effect of a targeted prevention program for childhood disruptive behavior on targeted factors (i.e., perspective taking and self-control) and associations between change in targeted factors and outcomes (i.e., aspects of disruptive behavior). The sample consisted of 173 children (*Mage* = 10.2 years) who were randomly assigned to an intervention condition (*n* = 70) or waitlist control condition (*n* = 103). Assessment took place at pre-, post- and follow-up measurements. For ethical considerations, follow-up data was not available for children on the waitlist. Findings revealed a direct intervention effect on self-control. From pre-test to follow-up, children who received the intervention improved in perspective taking and self-control. Moreover, improvements in self-control were associated with and predicted reductions in teacher-reported symptoms of oppositional defiant disorder. No associations were found between changes in perspective taking and disruptive behavior. These findings suggest that self-control may be an important target factor in reducing childhood disruptive behavior in targeted prevention.

## The Role of Perspective Taking and Self-Control in a Preventive Intervention Targeting Childhood Disruptive Behavior

Elementary school children with disruptive behavior problems are at increased risk for antisocial behavior and crime involvement (Kassing et al. 2019). In order to prevent problems later in life, it is essential to target childhood disruptive behavior with effective preventive interventions (Farrington et al. 2017). Although there are many effective preventive interventions to reduce disruptive behavior, overall treatment effects are only small to moderate (Weisz et al. 2017) and seem to vary widely in size (Farrington et al. 2017). To optimize the effectiveness of preventive interventions, it is needed to understand which characteristics of early targeted preventive interventions are specifically associated with reductions in disruptive behavior (Wilson et al. 2001).

Although the effectiveness of prevention programs has been studied quite extensively, most studies have focused on distal outcomes, rather than proximal factors (La Greca et al. 2009). Therefore, little is known about the specific factors that need to be targeted to effectively reduce childhood disruptive behavior (Dodge et al. 2013). Moreover, the relation between changes in targeted factors and outcomes is understudied (Lochman and Wells 2002; Weersing and Weisz 2002), since prevention research rarely focuses on the intervention’s theoretical framework (Hinshaw 2002). Testing the intervention’s rationale, in addition to its overall effect, provides insight into the specific factors that are responsible for behavioral change (Lochman and Wells 2002), and can thus be used to optimize treatment effects (Lochman et al. 2019).

Many cognitive behavioral treatments for childhood disruptive behavior are based on Crick and Dodge’s (1994) social information processing theory. According to this theory, social cognitive processes strongly influence individual’s (anti)social behavior. Social information needs to be processed following an orderly fashion of six steps: 1) encoding of social cues, 2) interpretation of social information, 3) response generation, 4) response decision, 5) enactment and 6) evaluation of enactment (Crick and Dodge 1994). Deficiencies in these steps may lead to impaired information-processing, which in turn may result in disruptive behavior (Crick and Dodge 1994; Arsenio 2010). Consecutively, interventions for disruptive behavior specifically target underlying social cognitive skills that are connected with specific deficiencies in social information processing steps.

A first factor that is often targeted in interventions for childhood disruptive behavior is *perspective taking,* which refers to the ability to understand others’ feelings and thoughts (Van Manen et al. 2009). Perspective taking is directly related to the first steps of the social information processing model: encoding and interpretation of social situations, as it helps to interpret a social situation correctly and to respond in an appropriate way (O’Kearney et al. 2017). Research shows that children with disruptive behavior problems tend to have difficulties with perspective taking, as they often neglect other’s thoughts and feelings (Van Manen et al. 2009) and are biased towards hostile attributions (Orobio de Castro et al. 2002). Thus, targeting perspective taking can enhance the first two information-processing steps.

Interventions and preventive programs such as the Coping Power Program, Self-control and Stay Cool Kids aim to improve perspective taking through the use of cognitive restructuring and role-play (Lochman and Wells 2002; Van Manen 2001; Stoltz et al. 2013). In these interventions, children are offered emotion education and are taught to assess and interpret their own and others’ feelings. Moreover, they learn to consider alternative interpretations for behaviors of others. The Coping Power Program, Self-control and Stay Cool Kids have been found to be effective in reducing disruptive behavior (Lochman and Wells 2002; Van Manen 2001; Stoltz et al. 2013). These interventions, however, consist of multiple treatment elements which target a diverse set of skills (Leijten et al. 2015). Since randomized controlled trials mainly examine the main effects of these interventions, relatively little is known about the interventions’ effect on targeted factors such as perspective taking.

The few studies that have specifically investigated the association between changes in perspective taking and changes in disruptive behavior indicate that improvements in perspective taking are related to reductions in disruptive behavior (Metropolitan Area Child Study Research Group 2007; O’Kearney et al. 2017; Stoltz et al. 2013; Lochman and Wells 2002). Still, these studies examined either overall social information processing or specific dimensions of perspective taking (e.g., emotion perspective taking, attributions or situational understanding), and did not specifically focus on multiple aspects of perspective taking. To our knowledge, only one study has focused on overall perspective taking. Van Manen (2006) found small support for the association between improvements in perspective taking and reductions in disruptive behavior. However, as the study focused on aggressive boys in clinical institutions, it is uncertain if, and how, changes in overall perspective taking are associated with changes in disruptive behavior in a targeted prevention setting.

A second factor that is often targeted in interventions for childhood disruptive behavior is *self-control,* which is defined here as the capacity to override a dominant stimulus-driven response with a weaker memory-driven response and is linked to the intrinsic regulation of behavior, emotion and cognition (Nigg 2017). Self-control is related to the last two steps of the social information processing model: enactment and evaluation of enactment and encompasses various ways of self-regulating behaviors such as delaying gratification and controlling impulses to execute goal-relevant responses (Nigg 2017). Children with behavior problems often have difficulties with self-control, as they may not consider consequences of their behavior, respond more impulsively and tend to evaluate the outcomes of disruptive behavior as favorable (Slaby and Guerra 1988; Van Manen 2001). Thus, by targeting self-control in interventions, the last two information-processing steps are expected to improve.

Interventions such as Self-control and Aggression Replacement Training (ART) aim to improve self-control through the use of anger control and problem-solving skills training (Van Manen 2001; Glick and Gibbs 2011). These interventions were found to be effective in reducing disruptive behavior (Van Manen 2001; Glick and Gibbs 2011). These findings suggest that improvements in self-control are related to reductions in disruptive behavior.

As with perspective taking, the relation between improvements in self-control and reductions in disruptive behavior is understudied (Weersing and Weisz 2002). The few studies that tested the relation between changes in self-control and changes in disruptive behavior indicated that improvements in self-control are associated with reductions in disruptive behavior (Guerra and Slaby 1990; Oostermeijer et al. 2016). Yet, aforementioned studies focused mainly on adolescents with a minimum age of 12 years. The only study that focused on elementary school children found an association between changes in self-control and changes in teacher- and parent-reported disruptive behavior (Van Manen 2006). Since few studies specifically tested this relation in children, more research is needed to obtain a better understanding of the need to address self-control to reduce childhood disruptive behavior.

The current study will zoom in on the theoretical rationale of a school-based social cognitive preventive intervention for disruptive children from disadvantaged neighborhoods: “Keep Cool … Start at School”. Since parents from low socioeconomic backgrounds are less likely to attend and complete interventions (Reyno and McGrath 2006), school-based early preventive interventions seem to be a good alternative, to parent-focused treatments, for disruptive children living in disadvantaged neighborhoods. “Keep Cool … Start at School” aims to improve perspective taking and self-control in order to reduce disruptive behavior. This intervention has been found to be effective in reducing disruptive behavior in an RCT-design using teacher reports on oppositional behavior problems and conduct problems, parent reports on total problem behavior and peer-reports on externalizing behavior. Directly after the intervention, an intervention effect was found for teacher-reported ODD and CD and parent-reported problem behavior (mean ES 0.31). At follow-up treatment, intervention effects were found for both teacher-, parent- and peer-reported disruptive behavior (mean ES 0.39; Liber et al. 2013). Notably, the effect of the intervention on perspective taking and self-control and the relation between changes in these targeted factors and reductions in disruptive behavior have not yet been addressed. The first aim of the current study, therefore, is to examine the intervention’s effect by taking two steps. First, it will be examined if children that received “Keep Cool … Start at School” show larger improvements in perspective taking and self-control, compared to children who did not receive the intervention. For ethical considerations, children from the waitlist received the intervention post-waitlist. Subsequently, we are only able to compare children who did and did not receive the intervention from pre-treatment to directly after treatment. Since previous studies found the strongest changes in perspective taking and self-control at follow-up (Van Manen 2006), we decided to take a second step in examining the intervention’s indirect effect. Thus, overall change in perspective taking and self-control will be examined from pre-test to follow-up intervention for children who received the intervention. The second aim of the study was to examine whether overall changes in perspective taking and self-control are related to changes in disruptive behavior. Based on aforementioned studies it is expected that the intervention will be effective in improving perspective taking and self-control directly after the intervention. Furthermore, it is expected that children will show larger improvements in perspective taking and self-control at follow-up compared to directly after the intervention. Additionally, it is expected that improvements in perspective taking and self-control will be associated with reductions in disruptive behavior over time.

## Method

### Participants

The sample consisted of 173 children (79% boys, 21% girls) aged between eight and 12 years (*M* = 10.27, *SD* = 1.19), their parents and their teachers. Children were included in three consecutive academic years starting in 2008–2009. In total, 70 children were assigned to the intervention condition and 103 children to the waitlist condition. Cluster randomization of classes resulted in unequal distributions, especially in year 3, and could therefore not be corrected. Socioeconomic status (SES) was categorized as low (*n* = 96; 56%), low to middle (*n* = 56; 32%) and high (*n* = 16; 9%). Fifty-seven children were Dutch (33%), seven children were of non-Dutch Western origin (4%), and 109 were (children of) non-Western immigrant non-Western immigrants (63%; i.e., Turkish, Moroccan, Surinamese, and Afghan). This sample was also used in a previous publication by (Liber et al. 2013) which reported the intervention’s effect on parent-, teacher- and peer-reported disruptive behavior. The current study elaborates on these findings by examining the effect of the intervention on perspective taking and self-control specifically, as well as their relation to reductions in disruptive behavior.

## Design

Cluster-randomization was used to randomly assign classes from 17 schools to the intervention condition or waitlist condition. For study year 1, six schools were contacted through the researchers’ network. For study year 2 and 3, school professionals of the participating schools in year 1 approached their networks which resulted in 11 participating schools. Prior to the study 20 numbers were assigned to sealed envelopes indicating randomization of grades 5–6 to the intervention condition and grades 7–8 to the waitlist condition or grades 7–8 to the intervention condition and grades 5–6 to the waitlist condition. Schools that consented to participate received a number and the corresponding envelope was opened. For nine schools, grades 5–6 were allocated to the intervention condition and grades 7–8 to the waitlist condition, and for eight schools, grades 5–6 were allocated to the waitlist condition while grades 7–8 were allocated to the intervention condition.

Assessment took place prior to the beginning of the first intervention wave (T1) and at the end of the first intervention wave (T2, 14 weeks after T1) for both children in the intervention and waitlist condition. For children in the intervention condition, T3 (15 weeks after T2) represents the follow-up measurement. Children in the waitlist condition received the intervention (i.e., intervention wave 2) between T2 and T3. Accordingly, for these children T3 represents the post-test measurement. Children within the waitlist condition were also measured at follow-up of the second intervention wave (T4; 17 weeks after T3). For ethical reasons, children in the waitlist condition received the second intervention wave directly after the first intervention wave ended. Subsequently, no follow-up data are available for children on the waitlist (i.e., who did not yet receive the intervention).

**Fig. 1:**
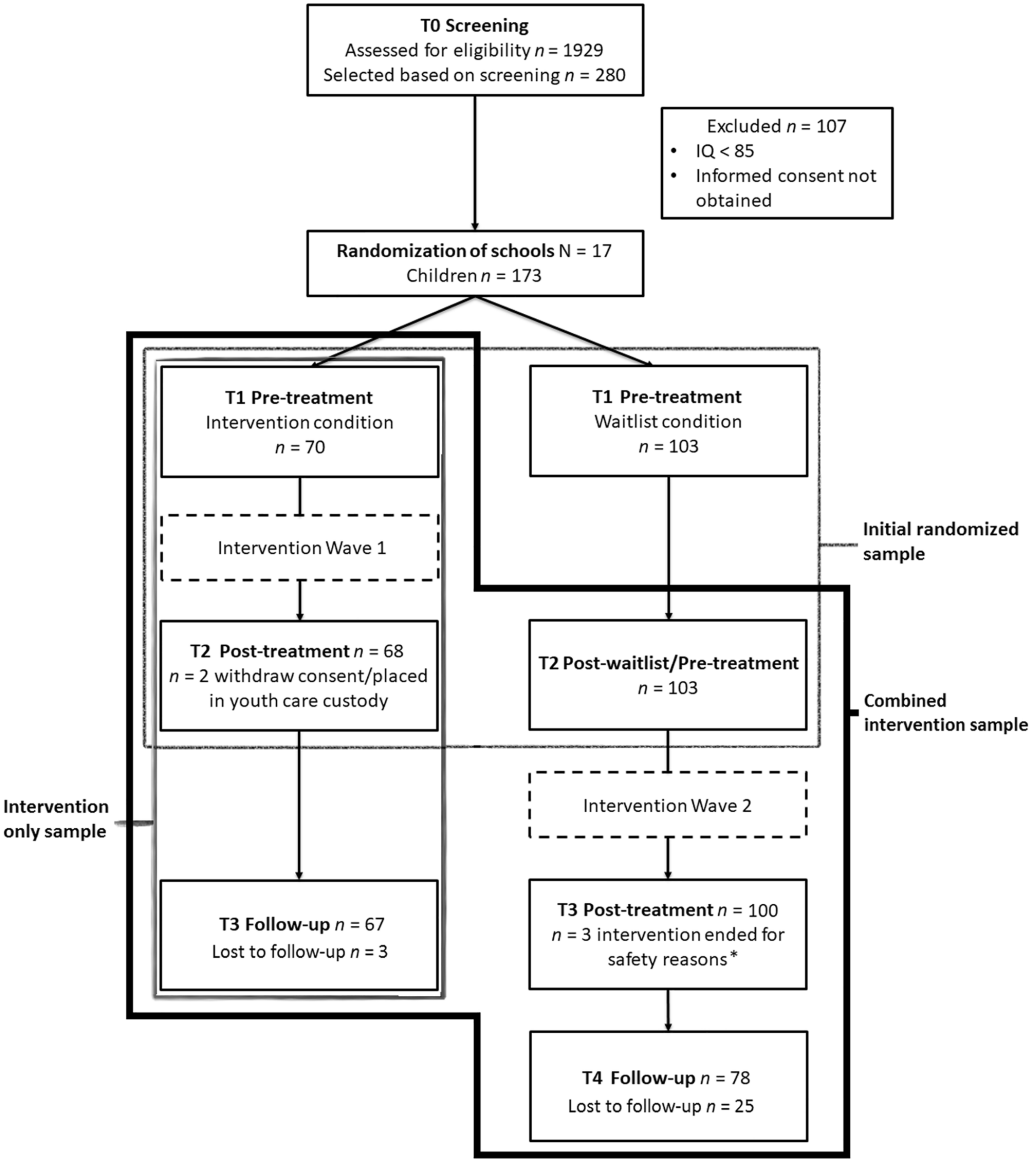
Flow Diagram and Content of the Study’s Sets of Data. *A safe, private and supportive environment could not be guaranteed during the training due to organizational difficulties (e.g., school in disarray, schoolboard dismissed.)

To maximize the power when testing our hypotheses, three sets of data were used in the current study. The first set of data was used to test the intervention’s direct effects on targeted mechanisms. This set of data was named the initial randomized sample and consisted of pre- (T1) and post-test measurements (T2) for the intervention and waitlist condition. The second set of data was used to examine change from pre-test to follow-up and the association between change in targeted mechanisms and outcomes. This set of data was named the combined sample because data from the intervention and waitlist condition were combined after the latter had received the intervention. As such, this set of data consisted of pre- (T1 = intervention wave 1/T2 = intervention wave 2), post- (T2/T3) and follow-up measurements (T3/T4). The third set of data was used to examine timing of change in the intervention group. This set was named the intervention only sample and consisted of the pre- (T1), post-(T2), and follow-up (T3) measurements of children in the intervention condition. See Fig. 1 for an overview of the participant’s flow and the samples of the current study.

## Procedure

Elementary schools from low or low-to-middle SES urban areas participated during three consecutive school years starting in September 2008 and ending in October 2011. Schools were only invited if they served a low SES population as reported by either the school principal or a representative of the school board. SES was categorized into three levels (i.e., low, middle, high) according to data from the Central Bureau of Statistics Netherlands (2010). This categorization includes 8 levels that were collapsed into 3 to facilitate interpretation. At the beginning of each school year, parents of children in grade five to eight (i.e., children aged eight to twelve years according to the Dutch educational system) received an information letter about the study. Parents were informed that they could object to school if they did not give permission to their child’s teacher to complete the List Global Screening (LGS; van Leeuwen and Bijl 2003). For each child, two teachers independently completed the LGS and their scores were then combined to select at-risk children. Based on this screening, 280 children were identified as at-risk (i.e., sum score of problem behavior ≥ 3 or sum score of problem behavior and risk of persistent problems ≥ 3). After screening and selection, 264 parents were invited by the school to sign informed consent for further participation. The parents of 16 children were not contacted to obtain consent because either the school objected (*n* = 8, one child was not sufficiently proficient in the Dutch language), or schools indicated that children had an IQ below 85 (*n* = 8). Parents of 10 children could not be reached, parents of 17 children declined participation, nine children already received mental health care, and for four children the reason for parents’ declining is unknown. Consent was thus obtained for 224 children. Assessment of eligibility revealed that 51 of these children had an estimated IQ below 85. An estimated IQ was obtained using either 2, 6 or all subtests of the Dutch version of the WISC-III (Thompson and LoBello 1994). All assessed children were sufficiently fluent in Dutch. Children with an estimated IQ < 85 were excluded from the study as it was expected that the cognitive content of the preventive intervention was too difficult for them. This resulted in a sample of 173 children (See Fig. 1). The study was approved by the Ethics Committee of the University of Amsterdam and was included in the Dutch Trial Register (NTR1352).

## Intervention

All children received the preventive intervention “Keep Cool … Start at School”, either in the first or second intervention wave. “Keep Cool … Start at School” is a manualized Cognitive Behavioral Treatment (CBT) program designed to reduce disruptive behavior in elementary school children from disadvantaged neighborhoods. The intervention was adapted from the *Self-Control* protocol by van Manen (2001) and consists of one individual session (15–20 min to set goals) and nine group sessions conducted by a principal trainer and a co-trainer at school. Group sessions take 90 min and are conducted in groups of three to eight children. Children are asked to complete assignments prior to sessions two to nine. The buildup of the nine group sessions follows the social information processing theory (Crick and Dodge 1994). During session 2, 3, 5 and 7, the intervention focuses on the improvement of perspective taking and the interpretation of social situations using cognitive behavioral techniques such as role-play, emotive education and modeling. During these sessions, children learn how to recognize emotions, how to relate with others and they learn about differences and mistakes in the interpretation of social situations. In session 1–6, 8 and 9, the intervention focuses on the improvement of self-control using role-play, positive reinforcement of adequate behavior and modeling. During these sessions, children learn how to control their anger and how to behave adequately. In addition, they learn about the consequences of their behavior. The intervention also included CBT-techniques aimed at emotion regulation. Elements that focused on emotion processes were included because behavior problems and emotion (dys)regulation seem to be related (Zeman et al. 2006). This is in line with the updated model of social information processing which stresses the importance of emotion processes (Lemerise and Arsenio 2000). In this way, the intervention aims to improve the child’s ability to control emotions, cognitions and behavior. For more information see the manual of “Keep Cool … Start at School” (De Boo & Liber 2014).

**Treatment Integrity and Attendance.** Weekly supervision meetings were held that included instructions, supplemental session information and discussions on the implementation of and adherence to the protocol. During the meetings, video fragments of the participating trainers were used. Thirteen trainers participated as principal trainer and 22 as co-trainer, which provided the intervention to 39 groups of children. In total, 146 children (84%) received all nine group sessions. Twenty-three children (13%) received seven or eight out of nine group sessions and one child (0.6%) received less than seven group sessions. The total number of sessions is unknown for one child. In addition, two out of 70 children in the intervention condition did not start or complete the preventive intervention, whereas three out of 103 children in the waitlist condition did not start or complete the preventive intervention. One child dropped out around session 7. Adherence to the treatment protocol was rated by life observers. The satisfactory mean adherence score was 0.86 (SD = 0.07, range 0.68-0.99, *n* = 169) as calculated for all available ratings per child. Ratings for all sessions were available for 146 children. For a more detailed description see (Liber et al. 2013).

## Measures

**Screening.** To identify at-risk children with symptoms of antisocial behavior, the List Global Screening (LGS; Van Leeuwen and Bijl 2003) was used. The LGS is a six-item screening questionnaire that assesses antisocial behavior in children. Items can be rated on a 3-point Likert scale ranging from *none/no significant problems* (0) to *significant problems* (2). The questionnaire includes three items addressing overt, covert, and oppositional problem behaviors (e.g., “To what extent does the child show oppositional defiant problem behavior?”). Item 4 measures the risk for persistent problems and item 5 and 6 reflect delays in educational development. A child was classified as at-risk (code 1) when the sum of the problem behavior items (i.e., item 1, 2 and 3; range 0 to 6) was 3 or greater or when the sum of the problem behavior items and the score for item 4 (risk for persistent problems) was 3 or bigger. Delays in educational development (item 5 and 6) augment the teachers’ at-risk estimation (item 4) with a maximum of 1 point. If these criteria were not met, the child was coded as ‘not at-risk’ (code 0). Validation research indicated good sensitivity (73%) and specificity (83%) in an ethnically diverse sample (758 children of which 170 were ‘at risk’; Van Leeuwen and Bijl 2003). Yule’s Y (measure for skewed distributions) was 0.60 which indicated sufficient interrater agreement. In the current study, the LGS was completed by two teachers for 1.929 children. Children were included in the study when both teachers rated the child as at-risk. The interrater agreement (Yule’s Y) was 0.61 among 151 teachers.

**Disruptive Behavior.** Disruptive behavior was rated by the teacher and the parent. Teachers completed the Disruptive Behavior Disorders Rating Scale (DBDRS; Dutch version, Oosterlaan et al. 2008). The DBDRS is a 42-item questionnaire which identifies symptoms of Oppositional Defiant Disorder (ODD; eight items), Conduct Disorder (CD; 16 items), attention problems (nine items) and hyperactivity/impulsivity. Items can be rated on a 4-point Likert scale ranging from *not at all* (0) to *very much* (3). The factor structure of the original version has been confirmed (Pillow et al. 1998). Cronbach’s alpha ranged from 0.81 to 0.95 and test–retest correlations ranged from 0.71 to 0.86 in the Dutch version (Oosterlaan et al. 2008), with exception of the CD scale which is negatively affected by low variance due to behaviors that are rare in nonclinical populations (e.g., mistreat of animals). The current study included the ODD scale (e.g., “Argues with adults”) and CD scale (e.g., “Has deliberately destroyed things from others”) to assess disruptive behavior. A mean score was constructed per scale, with a higher score indicating more symptoms of ODD or CD. Cronbach’s alpha ranged from 0.90 to 0.93 across measurements for the subscale ODD and from 0.74 to 0.75 for the subscale CD.

Parents completed the Strengths and Difficulties Questionnaire (SDQ; Dutch version, Van Widenfelt et al. 2003). The SDQ assesses children’s psychosocial problems and consists of 25 items. Items can be rated on a 3-point Likert scale ranging from *not true* (0) to *certainly true* (2). For the parent-reported total difficulties scale, Cronbach’s alpha was 0.81 in a Dutch sample of 285 parents (Van Widenfelt et al. 2003). Furthermore, results indicated sufficient inter-informant correlations between parents and teachers (*r* = 0.52) and between parents and youth (*r* = 0.47; Van Widenfelt et al. 2003). The present study included the Total Difficulties scale (20 items) to assess problem behavior. A mean score was constructed, with higher scores indicating more problem behavior. Cronbach’s alpha ranged from 0.78 to 0.83 across measurement times, except at follow-up for the waitlist condition (α = 0.62, *n* = 21).

**Perspective Taking.** Perspective taking was assessed by the Social Cognitive Skills Test (SCST; Van Manen et al. 2009). The SCST assesses social cognitive skills in children aged 4 to 12 years using seven short stories with corresponding pictures. The stories involve a social situation in which the child is confronted with a problem. The SCST theorizes that the child’s social cognitive development follows a sequence of eight social cognitive skills: 1) identification of the other’s perspective, 2) discrimination between different perspectives, 3) differentiation, 4) comparing, 5) empathizing, 6) relating between perspectives, 7) coordinating and 8) discounting of others’ perspectives (Van Manen et al. 2009). Each story assesses the eight social cognitive skills by eight systematic questions that tap into these skills (e.g., “How does the boy feel on picture 1?”). The stories were told by the experimenter and answers were rated as follows: *incorrect answer* (0), *partly correct answer or correct answer after additional question* (1), *correct answer* (3). The factor structure of the SCST has been confirmed as one general factor (total score) and as eight subdomains. Cronbach’s alpha was 0.96 in a sample of 2264 Dutch children and test–retest reliability ranged from 0.82 to 0.85 (Van Manen et al. 2009). In the current study, a mean score of all items was included in which higher scores indicate higher levels of perspective taking. It was decided to use a total mean score, since this was expected to provide a more reliable examination of overall change in all subdomains related to perspective taking, instead of a specific subdomain (Van Manen 2001). Cronbach’s alpha ranged from 0.82 to 0.87 across measurement times. Responses were rated by master-level students. A sample of 29 video-taped assessments was recoded by 4 trained pre-master students which revealed sufficient interrater agreement (ICC = 0.79).

**Self-Control.** Self-control was rated by the teacher using the Self-Control Rating Scale (SCRS; Kendall and Wilcox 1979). The SCRS measures both the behavioral (response inhibition) and cognitive components (problem-solving) of self-control and consists of 33 items (e.g., "Does the child stick to what he or she is doing until he or she is finished with it?"). Items can be rated on a 7-point rating scale ranging from *maximum self-control* (1) to *maximum impulsivity* (7). A confirmative factor analysis indicated a one-factor model in a sample of 110 US children between 8 and 12 years. Chronbach’s alpha was 0.98 and the test–retest reliability was 0.84 (Kendall and Wilcox 1979). In the present study, a mean score of all items was included. As items have been recoded, higher scores indicate higher levels of self-control. Cronbach’s alpha ranged from 0.94 to 0.97 across measurement times.

## Data Analytic Strategy

An examination of missing data revealed that the teacher of one child and the parents of 11 children did not report on disruptive behavior on all measurement times. Twenty-eight children missed follow-up data. This was predominantly the case for children that received the second intervention wave (*n* = 25, i.e., waitlist condition; T4). In most of these cases, children dropped out as they changed schools after summer. Attrition analyses were performed to compare the samples with complete (*n* = 145) and incomplete data (*n* = 28). Results indicated that the samples did not significantly differ regarding baseline levels of disruptive behavior, perspective taking, self-control, child’s gender, ethnicity or SES.

All data were analyzed using Mplus (Version 8; Muthén and Muthén 2017). Since children were nested within intervention groups, the hierarchical structure of the data was taken into account (intra-class correlation ranged between 0.04 and 0.15 in initial randomized sample and between 0.05 and 0.34 in combined intervention sample). As the number of clusters was not sufficient to conduct multilevel analysis (i.e., 13 instead of 20 clusters; Muthén 2005), mean-centered dummy variables of intervention groups were included as covariates. To reach model parsimony, insignificant effects of specific intervention groups were fixed to zero. Little’s Missing Completely at Random (MCAR) test indicated that data was MCAR in both samples (normed χ^2^ (χ^2^/*df*) was 1.03 in initial randomized sample and 1.01 in combined intervention sample; Little 1988). Therefore, maximum likelihood estimation (ML) was used to estimate missing data. The model fit was tested using the Comparative Fit Index (CFI) and the Root Mean Squared Error of Approximation (RMSEA). The cutoff criteria by Little (2013) were used to interpret fit indices. Good model fit was obtained when CFI was equal or above 0.95 and when RMSEA was equal or below 0.05.

**Initial Randomized Sample.** To investigate the intervention’s direct effect on perspective taking and self-control from pre- to post-test, the initial randomized sample was used. First, it was examined whether there were differences between the two conditions at baseline. Subsequently, between-group differences (intervention versus waitlist) in change were tested using Structural Equation Modeling (SEM) path analyses. To examine intervention effects, post-test scores of perspective taking and self-control were regressed on pre-test scores and condition (0 = *waitlist*, 1 = *intervention*). To report on effect sizes, Cohen’s *d* was computed using a two-step approach, as recommended by Feingold (2019). First, the pooled standard deviation was calculated (√ (SD^2 ^_waitlist_ + SD^2^ _intervention_) / 2). Second, Cohen’s *d* was calculated using the Model constraint option in Mplus. Values of *d* = 0.20 were considered as small effects, 0.50 as medium and 0.80 as large effects (Cohen 1992).

**Combined Treatment Sample.** To examine change in perspective taking and self-control from pre-test to follow-up, the combined intervention sample was used in which pre-, post- and follow-up measurements of the first and second intervention wave were merged (See design section for a more detailed description). The trajectories of perspective taking and self-control were identified and modeled with univariate latent growth models. The linear model was examined using an *intercept* (with factor loadings of three observed variables, corresponding to three measurements, set at 1) and a *slope factor* (with the factor loadings set at 0, 1 and 2, since there were approximately equal time intervals between measurements). Slope factors were estimated freely for one of the occasions when a linear model did not fit the data. To reach model parsimony, univariate models were optimized by fixing insignificant effects of intervention groups to zero. The standardized estimates were interpreted as correlations to report on effect sizes: values less than 0.10 indicate small effects, around 0.30 indicate medium effects, and around 0.50 indicate large effects (Cohen 1988).

To examine the association between changes in perspective taking and self-control and changes in disruptive behavior, univariate latent growth models were also conducted for parent-reported problem behavior, teacher-reported ODD and teacher-reported CD following the same procedure as mentioned above. In a second step, bivariate latent growth models were conducted in which the best fitting (optimized) univariate models were combined. Six models were estimated either with a combination of perspective taking and one of the different measures of disruptive behavior or with self-control and one of the measures of disruptive behavior. Growth parameters of perspective taking, self-control and disruptive behavior were estimated simultaneously and were allowed to correlate. In each model, it was examined whether there was a correlation between the constructs’ slopes.

**Intervention Only Sample.** The timing of change in perspective taking and self-control and change in disruptive behavior for children in the intervention condition was tested using SEM path analyses. First, residual change scores were calculated in SPSS Statistics 26, in order to represent change in perspective taking and self-control from pre- to post-test and change in disruptive behavior from pre-test to follow-up. Second, change scores of disruptive behavior were regressed on change scores of perspective taking and self-control in Mplus.

## Results

### Preliminary Analyses

Chi-square tests and Analysis of Variance (ANOVA) were performed to examine whether relevant child’s characteristics were equally distributed across conditions. One significant between-group difference was found on SES, χ^2^(2) = 7.28, *p* = 0.026. Within the intervention condition, the number of children from low SES backgrounds (44%) and from low to middle SES backgrounds (39%) was approximately equal, whereas in the waitlist condition a larger percentage of children was from low SES backgrounds (63%) compared to low to middle SES backgrounds (28%). In both conditions, the smallest percentage of children where from high SES backgrounds. No significant between-group differences were found for all other background variables (i.e., child’s gender, age and ethnicity) and for baseline levels of perspective-taking, self-control and disruptive behavior. These results indicate that randomization succeeded.

## Intervention Effects on Perspective Taking and Self-Control from Pre- to Post-Test

Means and standard deviations of all measurements are presented in Table 1. In addition, correlations between constructs measured at pre- and post-test are included in Table 2. Results showed that there was no significant intervention effect for perspective taking, *B* = 0.05, 95% CI [-0.10,0.20], *p* = 0.505. However, a significant intervention effect was found for self-control, *B* = 0.23, 95% CI [0.00,0.45], *p* = 0.048, *d* = 0.23, indicating that “Keep Cool … Start at School” improved self-control at post-test.

**Table 1:** Means and Standard Deviations of Pre-, Post- and Follow-Up Measurements

Outcome							
Intervention condition (*n* = 70)				Waitlist condition (*n* = 103)			
	Pre-treatment (T1)	Post-treatment (T2)	Follow-up (T3)	Pre-test (T1)	Post-waitlist / pre-treatment (T2)	Post-treatment (T3)	Follow-up (T4)
Perspective taking	2.3 (0.4)	2.3 (0.3)	2.4 (0.4)	2.3 (0.4)	2.3 (0.3)	2.4 (0.4)	2.4 (0.4)
Self-control	3.6 (0.9)	3.9 (0.9)	3.9 (0.9)	3.8 (1.0)	3.8 (1.0)	4.0 (1.0)	4.0 (1.1)
Problem behavior P	0.7 (0.3)	0.5 (0.3)	0.5 (0.3)	0.7 (0.3)	0.6 (0.3)	0.6 (0.3)	0.6 (0.2)
ODD T	1.2 (0.6)	0.9 (0.6)	1.0 (0.6)	1.1 (0.7)	1.1 (0.7)	0.9 (0.7)	0.8 (0.7)
CD T	0.2 (0.2)	0.1 (0.2)	0.1 (0.2)	0.2 (0.2)	0.2 (0.2)	0.2 (0.2)	0.1 (0.1)

*P* parent-reported, *T* teacher reported. For all variables, higher scores indicate higher levels of that specific construct

**Table 2:** Correlations between Constructs at Pre-Test and Post-Test for the Intervention and Waitlist Condition

Correlations										
	PT T1	SC T1	PPB T1	ODD T1	CD T1	PT T2	SC T2	PPB T2	ODD T2	CD T2
PT T1	1	0.01	0.16	0.12	0.07	0.42**	0.05	-0.14		-0.10
SC T1	0.06	1	-0.15	-0.55**	-0.42**	-0.02	0.75**	-0.03	-0.32**	-0.24*
PPB T1	0.00	0.09	1	0.24*	0.15	-0.26*	-0.23*	0.76**	0.24*	0.40**
ODD T1	0.05	-0.56**	0.04	1	0.55**	-0.04	-0.51**	0.09	0.73**	0.41**
CD T1	0.11	-0.38**	0.18	0.62**	1	-0.14	-0.33**	0.00	0.40**	0.43**
PT T2	0.56**	0.08	0.16	-0.13	-0.04	1	0.02	0.04	-0.61	-0.29**
SC T2	0.09	0.73**	-0.03	-0.42**	-0.30*	0.16	1	-0.21	-0.51**	-0.39**
PPB T2	-0.11	0.10	0.65**	-0.07	0.07	-0.02	-0.10	1	0.24*	0.36**
ODD T2	0.04	-0.30*	0.06	0.62**	0.32*	0.07	-0.48**	0.11	1	0.60**
CD T2	0.08	-0.25*	0.06	0.40**	0.55**	0.04	-0.40**	-0.01	0.63**	1

Correlations for intervention condition are in the lower left corner, correlations for waitlist condition are in the upper right corner

*PT* perspective taking, *SC* self-control, *PPB* parent-reported problem behavior, *ODD* teacher-reported ODD, *CD* teacher-reported CD

*p <0.05, **p < 0.01

## Evaluating (Associations in) Change from Pre-Test to Follow-up Intervention

**Overall Change from Pre-Test to Follow-up Intervention.** For this analysis, data from the intervention and waitlist condition were combined (See design section for a more detailed description). Univariate latent growth models were conducted to analyze change within perspective taking and self-control from pre-test to 3-month follow-up. For perspective taking and self-control, a linear growth model that was optimized (i.e., all insignificant paths of therapy group fixed to zero) showed acceptable to good fit. Fit statistics and slope’s estimates of the final univariate models are shown in Table 3. Results indicated that children who received the intervention either in the first or second intervention wave significantly improved in perspective taking and self-control from pre-test to 3-month follow-up.

**Table 3:** Fit Statistics and Parameter Estimates for Univariate LGMs and Bivariate LGMs Conducted with Combined Treatment Sample

Model	*n*	χ^2^ (*df*)	*p*	CFI	RMSEA	*B*_slope_	*SE*	β_slope_	*p*	σ_slope_		*p*
*Univariate LGMs*												
Perspective taking	172	31.61 (30)	0.386	0.98	0.02	0.11	0.03	0.96	< 0.001	0.01	0.760	
Self-control	172	50.41 (35)	0.044	0.92	0.05	0.14	0.04	0.34	0.002	0.16	0.012	
Problem behavior P	144	36.48 (34)	0.354	0.95	0.02	-0.09	0.03	-0.39	0.003	0.05	0.071	
ODD T	172	33.99 (28)	0.201	0.96	0.04	-0.11	0.03	-0.59	< 0.001	0.01	0.730	
CD T^a^	171	41.48 (36)	0.244	0.89	0.03	-0.02	0.03	-0.09	0.367	0.06	< 0.001	
	*n*	χ^2^ (*df*)	*p*	CFI	RMSEA	*r*_ss_	SE	*p*				
*Bivariate LGMs*												
Perspective taking & Problem behavior P	173	69.74 (72)	0.554	1.00	0.00	0.00	0.01	0.915				
Perspective taking & ODD T	172	66.86 (63)	0.346	0.98	0.02	0.01	0.01	0.175				
Perspective taking & CD T^a^	172	77.74 (71)	0.276	0.95	0.02	-0.01	0.01	0.219				
Self-control & Problem behavior P	173	91.60 (77)	0.123	0.94	0.03	-0.01	0.02	0.636				
Self-control & ODD T	172	107.34 (68)	0.002	0.91	0.06	-0.09	0.02	< 0.001				
Self-control & CD T^a^	172	91.57 (76)	0.108	0.94	0.04	-0.04	0.02	0.011				

*P* parent-reported, *T* teacher-reported, *CFI* Comparative Fit Index, *RMSEA* Root Mean Square Error of Approximation

^a^Revised model, slope factor loading of T3 is freely estimated

**Correlated Change between Targeted Factors and Disruptive Behavior.** Before correlated change was examined, univariate latent growth models were conducted for disruptive behavior. For teacher-reported CD, an optimized linear growth model did not fit the data, χ^2^(36) = 56.17, *p* = 0.017, AIC = 32.24, BIC = 60.51, CFI = 0.59, RMSEA = 0.06. An optimized model in which the slope factor loading of the follow-up measurement (T3) was freely estimated showed a better fit (i.e., mediocre fit), ΔAIC = -14.69, ΔBIC = -14.69. For teacher-reported ODD and parent-reported problem behavior, an optimized linear model showed acceptable to good fit. See Table 3 for the fit statistics of the final univariate models.

Next, bivariate growth models were conducted. Fit statistics of the final bivariate latent growth models and slope-slope correlation coefficients are also included in Table 3. All models with perspective taking and one of the constructs of disruptive behavior showed good fit. The models with self-control showed acceptable fit. Correlations between the slopes of perspective taking and the slopes of parent-reported problem behavior and teacher-reported ODD and CD were non-significant. This indicates that from pre-test to 3-month follow-up, improvements in perspective taking are not associated with reductions in parent-reported problem behavior and teacher-reported ODD and CD. Moreover, no significant correlations were found between the slopes of self-control and parent-reported problem behavior. However, significant weak negative slope-slope correlations were found between self-control and teacher-reported ODD and teacher-reported CD. These results indicate that from pre-test to 3-month follow-up, improvements in self-control are weakly associated with reductions in teacher-reported ODD and CD, but not with reductions in parent-reported problem behavior.

## Timing of Change

**Change in Perspective Taking and Disruptive Behavior.** No significant effects were found for change in perspective taking from pre-test to post-test on change in teacher-reported ODD from pre-test to follow-up, *B* = 0.18, 95% CI [-0.07,0.42], *p* = 0.161. Also, no effect was found for change in perspective taking from pre-test to post-test on change in teacher-reported CD from pre-test to follow-up, *B* = 0.09, 95% CI [-0.18,0.35], *p* = 0.525. Finally, no significant effect was found for change in perspective taking from pre-test to post-test or change in parent-reported problem behavior from pre-test to follow-up, *B* = -0.22, 95% CI [-0.33,0.29], *p* = 0.888.

**Change in Self-Control and Disruptive Behavior.** Results showed a significant effect of change in self-control from pre-test to post-test on change in teacher-reported ODD from pre-test to follow-up, *B* = -0.27, 95% CI [-0.50,-0.05], *p* = 0.018. Change in self-control from pre-test to post-test did have a significant effect on change in teacher-reported CD from pre-test to follow-up, *B* = -0.38, 95% CI [-0.59,-0.17], *p* < 0.001. No significant effects were found for pre-post change in self-control on change in parent-reported problem behavior from pre-test to follow-up, *B* = -0.22, 95% CI [-0.56,-0.12], *p* = 0.205.

## Discussion

The goal of the current study was to zoom in on the theoretical rationale of a social cognitive preventive intervention for disruptive children from disadvantaged neighborhoods: “Keep Cool … Start at School”. An earlier publication indicated that the intervention was effective in reducing disruptive behavior (Liber et al. 2013). The current study elaborated on these findings by examining the effects of the intervention on the targeted factors perspective taking and self-control, and whether changes in these targeted factors were related to changes in disruptive behavior. Our findings revealed a significant direct effect of the intervention on self-control. In addition, from pre-test to follow-up, children who received the intervention improved in perspective taking and self-control and reduced in disruptive behavior. Improvements in self-control were significantly associated with reductions in teacher-reported disruptive behavior, whereas changes in perspective taking and disruptive behavior were not related over time. In line with this finding, we found that change in self-control after intervention termination predicted change in disruptive behavior at follow-up. This relation was not found for change in perspective taking and disruptive behavior.

Based on the findings, it can be concluded that “Keep Cool … Start at School” has a small direct effect on the targeted factor self-control, immediately after intervention termination. This means that, in accordance with the theoretical rationale of the intervention, children who are at-risk for disruptive behavior problems, increase in their self-control skills. Contrary to our expectations, children who received the intervention did not differ from children in the waitlist control condition in perspective taking. This indicates that there may not be a direct effect of the intervention on the targeted skill perspective taking.

This finding is not in line with previous studies, in which it was found that children who participated in cognitive behavioral (preventive) interventions similar to “Keep Cool … Start at School” did improve in perspective taking skills (Lochman and Wells 2002; Van Manen 2006; Stoltz et al. 2013). However, previous studies examined the effect of the intervention on children’s changes in perspective taking from prior to the intervention to one year after the intervention, rather than immediately after intervention termination. A possible explanation for this difference in findings could be that children’s changes in perspective taking skills do not occur directly after the intervention but are slowly developing over time.

The current study’s findings, with regard to change over time, are in accordance with this explanation, since we found that children who received “Keep Cool … Start at School” either at the beginning of the study or after a waitlist period, showed medium to large improvements in perspective taking from prior to the intervention to three months after intervention termination. Thus, it may be that children do indeed develop enhanced perspective taking skills after receiving the intervention, but that these increases are gradual, rather than immediate. Indeed, perspective taking, and related social information processing are complex processes that depend on earlier life experiences. Based on these experiences, children may develop stable social-cognitive processing patterns (Dodge et al. 2013). Due to its stability and experience-dependency, changes in perspective taking may be delayed. Previous studies with a similar population indeed found that children show larger changes in perspective taking, self-control and disruptive behavior, after at least three months post intervention termination than directly after treatment termination (Van Manen 2006; Liber et al. 2013).

The current study also found evidence for an increase over time in self-control. Importantly, as expected, the increase in self-control was associated with the decrease in teacher-reported disruptive behavior. Thus, children who showed, on average, steeper increases in self-control skills, also showed larger decreases in disruptive behavior. Moreover, results indicated that change in self-control after intervention termination predicts overall change in teacher-reported disruptive behavior. Contrary to our expectations, improvements in perspective taking skills were not associated with and could not predict reductions in disruptive behavior. Overall, these findings suggest that self-control may be an important target factor for change in disruptive behavior in prevention programs for elementary school children with disruptive behavior problems. This finding was in line with our expectations, as previous studies showed that there was a small to medium relation between children’s improvements in self-control and reductions in disruptive behavior (Van Manen 2006; Guerra and Slaby 1990; Oostermeijer et al. 2016). The merely weak association between improvements in self-control and reductions in disruptive behavior, could reflect that “Keep Cool … Start at School” addresses multiple change processes due to the use of different CBT techniques. In this way, change in disruptive behavior could not be explained by one dominant factor (e.g., self-control) and is an accumulation of multiple changes (Muris et al. 2005).

The difference in findings for self-control and perspective-taking could potentially be explained by the relatively low dosage of group sessions in “Keep Cool … Start at School” that specifically focused on perspective taking (i.e., only 4 out of 9 sessions compared to 8 out of 9 sessions on self-control; Foster 2003). Thus, the number of sessions may not have been sufficient to improve typical deficits in perspective taking that were related to disruptive behavior. Remarkably, however, our findings did indicate that children showed large improvements in perspective taking. It is therefore also possible that the measure used to assess perspective taking (i.e., Social Cognitive Skills Test; SCST) was not sensitive enough to sufficiently test changes in perspective taking skills. Muris et al. (2005) have suggested that the SCST is less suitable to assess typical deficits and distortions in perspective taking as this measure mainly focuses on the child’s development through social cognitive phases. For future research it is therefore recommended to explicitly focus on skill-based improvements in perspective taking by using experimental measurements such as video vignettes (i.e., the Social Information Processing Test (SIVT in Dutch; Van Rest et al. 2019) or Virtual Reality.

It should be noted that no associations were found between change in targeted factors and change in parent-reported problem behavior in the current study. This might be explained by the fact that more than half of the parents did not complete the questionnaire at post-test. The low parent completion rate is expected to result from population characteristics; we focused on children and parents from low SES, who, overall, seem to be exposed to bigger and more frequent life stressors (Gallo and Matthews 2003) and may therefore be more difficult to involve (Reyno and McGrath 2006). Notably, the low SES population could also play a role in explaining the differences in associations between change in self-control, perspective taking and disruptive behavior. Children with disruptive behavior problems from low income families are more likely to have difficulties with emotion dysregulation due to harsh parenting and observations of violence in their families or communities (Huaqing Qi and Kaiser 2003). This suggests that children from low SES backgrounds may show disruptive behavior due to low levels of self-control that are maintained by negative environmental influences (e.g., violent role models). Consequently, improvements in perspective taking may not be specifically related to reductions in disruptive behavior. It might be interesting to further examine these associations within samples from different backgrounds for a more in-depth understanding of factors that need to be targeted to reduce disruptive behavior.

## Strengths and Limitations

Strengths of the current study were that disruptive behavior was assessed from a multi-informant perspective (i.e., parents and teachers). In addition, the intervention was completed by more than 95% of the children. Although this study brings important implications for preventive interventions targeting disruptive behavior in children from disadvantaged neighborhoods, some limitations need to be addressed. First, it should be noted that the current’s study’s findings with regard to (associations in) change over time cannot be attributed with certainty to the intervention as, for ethical reasons, follow-up assessments of the waitlist control group could not be included. Children in the waitlist condition therefore also received the intervention, but later in time. An advantage of this approach is that change over time was examined for a larger sample, but this also meant that we could not compare the change over time in targeted skills and outcomes with a group of children who did not receive the intervention. We were, therefore, not able to rule out the alternative explanation that observed changes were due to maturation, rather than intervention effects. In a randomized controlled trial with a follow-up measure of children who had not received the intervention, a mediation analysis could have further assessed whether changes in targeted factors *precede* changes in outcomes. An alternative design would be a randomized micro-trial (Leijten et al. 2015), in which children are, for example, exposed to *either* self-control or perspective taking training. A second limitation was that, although adherence ratings were satisfactory, there was variability in adherence (range 0.69-0.99). The source of this variability is unknown; i.e., trainers ‘not adhering’ or in-session challenging behavior that needed to be addressed. Thus, there may be variability in the treatment content that children received. Third, children from different SES backgrounds were not equally distributed across conditions, which may have influenced the results.

## Implications

Despite these limitations, the current study suggests that disruptive behavior in elementary school children from disadvantaged neighborhoods can be effectively reduced by improving self-control skills. These findings can be used to optimize preventive interventions. Specifically, this study suggests that by improving self-control, a potential life-course persistent pathway of antisocial behavior may be prevented in childhood. Therefore, an enhanced focus on self-control, may potentially enhance treatment effectiveness. Thus, although further research is needed, our findings provide a better understanding of which mechanisms may contribute to positive effects of cognitive behavioral preventive interventions.

## Data Availability

The datasets analyzed during the current study are available from the corresponding author upon reasonable request.

## Code Availability

The syntax that was used to analyze the data is available from the corresponding author upon reasonable request.
